# A Pilot Study Examining the Experience of Veterinary Telehealth in an Underserved Population Through a University Program Integrating Veterinary Students

**DOI:** 10.3389/fvets.2022.871928

**Published:** 2022-04-25

**Authors:** Lauren Lundahl, Lauren Powell, Chelsea L. Reinhard, Eleni Healey, Brittany Watson

**Affiliations:** ^1^School of Veterinary Medicine, University of Pennsylvania, Philadelphia, PA, United States; ^2^Philadelphia Animal Welfare Society, Philadelphia, PA, United States

**Keywords:** access to care, telehealth, barriers to veterinary care, veterinary student, veterinary education, human-animal bond

## Abstract

Cost and transportation are two commonly cited barriers to accessing health care in both human and veterinary medicine within underserved communities. While human medicine has utilized telehealth as a means of breaking down this barrier, limited research exists to describe its use in veterinary medicine. The Pets for Life (PFL) program has partnered with the Penn Vet Shelter Medicine Program to provide veterinary appointments to clients, at no cost to the client, in underserved zip codes through virtual telehealth visits. These visits incorporated veterinary students as part of their clinical rotations through a service learning based model. Between January and August 2021, 31 PFL clients and nine veterinary students completed surveys to describe the role of telehealth in addressing barriers to accessing veterinary care, their perceptions of telehealth appointments, the human-animal bond, and changes in veterinary student empathy. PFL clients completed the survey immediately following their telehealth appointment, and veterinary students completed surveys prior to and following their participation in the PFL appointments during the rotation. Nearly 25% of clients reported that they would not have been able to secure transportation and 58% reported they would not have been able to afford an appointment at an in-person veterinary clinic. The population of clients who responded that cost was a significant barrier to accessing care did not entirely overlap with those who responded that transportation was a significant barrier to accessing care, indicating support for the use of telehealth in providing an alternative modality to address transportation challenges as a barrier to accessing veterinary care. Additional data suggests that both client and student experience was overwhelmingly positive, providing support for further service learning initiatives in veterinary student education. Further research is warranted to continue to assess the emerging role of telehealth in improving veterinary care for underserved communities.

## Introduction

Social and economic factors, including access to food and transportation, housing status, and educational attainment, contribute to up to 40% of human health outcomes (1). Individuals who lack security in these social and economic factors subsequently experience poorer health outcomes that can ultimately negatively affect both length and quality of life. Similar barriers can also prevent underserved pet owners from accessing veterinary care. Although there is overall a lack of literature regarding veterinary care in underserved communities, the most commonly identified barriers to care include cost, accessibility of care, lack of veterinarian-client communication, culture/language, and lack of client education (2).

One initiative that has tried to address these barriers to human health care in underserved communities includes the use of telemedicine and telehealth. Telemedicine is defined by the World Health Organization as

The delivery of health care services, where distance is a critical factor, by all health care professionals using information and communication technologies for the exchange of valid information for diagnosis, treatment, and prevention of disease and injuries, research and evaluation, and for the continuing education of health care providers, all in the interests of advancing the health of individuals and their communities [(3), p. 9].

Telemedicine is further differentiated from telehealth as being, “restricted to service delivery by physicians only, and the latter signifying services provided by health professionals in general, including nurses, pharmacists, and others” [(3), p. 9]. The broader definition of telehealth is utilized for the remainder of this paper. A review of the literature regarding telehealth in developing countries shows that telehealth can improve access to quality healthcare and can even allow patients to seek earlier treatment with better continuity of care, especially for those with chronic conditions (3). Additionally, recent studies that examine the use of telehealth specifically during the SARS-CoV-2 pandemic show that the use of televisits are an important resource for increasing access to care, specifically for non-surgical specialties (4). Research on the use of telehealth in veterinary medicine specifically is limited, however published data supports similar advantages to pet owners in terms of increased access to care and overall positive experiences (5).

Telehealth has also been described as a means for providing educational opportunities for medical students. A mixed-methods review of literature regarding telehealth training in medical education indicates that it has been integrated into lessons, ethics case studies, clinical rotations, and teleassessments, to provide a valuable experience for students (6). However, its use in human medical education remains relatively limited and further incorporation of this emerging platform is indicated (7).

Current methods for providing client communication education for veterinary students include staged interactions, small group communication teaching, peer assisted learning, and evaluation of recorded authentic client interactions (8–11). A unique approach to client communication education includes the use of service learning. Service learning focuses on reflecting upon service based community experiences in a reciprocal nature in order to benefit all participants (12). A recent study in the veterinary field has shown that students who have participated in a low cost clinic that promotes service learning have positive experiences that provide valuable learning opportunities while improving access to care for pet owners (13). There is currently very little research on the impact of telehealth in veterinary education, and no known studies on its use within the context of underserved communities. The purpose of this study was to evaluate the experience of veterinary telehealth for underserved pet owners and the ability of telehealth to eliminate barriers to veterinary care within underserved communities. A secondary aim was to describe veterinary students' perceptions of telehealth and changes in student empathy following participation in a service learning veterinary telehealth rotation.

## Materials and Methods

### Pets for Life Program

The purpose of the Pets for Life (PFL) programming of the Humane Society of the United States is to provide pet resources to members of underserved communities through positive and long-term relationships in the context of door-to-door community outreach (14). PFL outlines distinct initiatives that allow their organization to address potential barriers to pet ownership. One of these initiatives is direct care, which utilizes relationships built through an established community liaison to schedule at-home veterinary visits to clients and their animals in specific neighborhoods of Philadelphia, at no cost to the client (14).

The Penn Vet Shelter Medicine Program has partnered with PFL to offer medical and surgical services for over 8 years. Faculty, interns, and supervised students through that time period have offered varied programs including vaccine clinics, spay-neuter surgery, and at-home visits for patients. The Penn Vet Shelter Medicine Program and PFL has had a continued interest in developing a telehealth component to public outreach programs to help alleviate some challenges in accessible care as indicated in human literature (3). The SARS-CoV-2 pandemic accelerated this development process as the team recognized that at-home visits were a high risk for disease transmission but wanted to continue to provide services to the community. The Penn Vet Shelter Medicine Program hosts an internship program for veterinary graduates, which is offered through the Veterinary Internship and Residency Matching Program (VIRMP) (15). This internship program requires completion of a community-directed project, and in 2020, the project focused on the development of a new telehealth service for PFL clients. These appointments are seen by interns, faculty, and veterinary students during the shelter medicine clinical rotation. As part of the program's development, assessment of the program's impact for clients and their pets was integrated. Since this service was being utilized in the shelter medicine rotation during the SARS-CoV-2 pandemic for student experiences, the pilot evaluation also included evaluating the perceptions of telehealth and clients for student education.

### PFL Appointment Structure

The PFL program independently scheduled individuals for appointments appropriate for telehealth as designated by the Penn Vet Shelter Medicine team. A community liaison, an employee of PFL, was in charge of communicating with clients in Spanish, troubleshooting technology, and scheduling appointments. The liaison participated in appointments individually through the virtual platform. At least one PFL community liaison was in attendance during at least a portion of each appointment. All appointments were conducted via a virtual video chat platform (16), and each appointment was scheduled for a 1 h time slot. Approximately three appointments were scheduled per day. The Penn Vet Shelter Medicine Program faculty and staff hosted appointments once per week. Students were present for these appointments twice a month. The remainder of the appointments were hosted by the program faculty and/or intern only.

These appointments ranged from general health consultations to non-life threatening sick appointments. For each scheduled appointment, the veterinary team and veterinary students would sign on to the virtual platform along with the client and community liaison. Each person would join the appointment individually through their personal device. The veterinary team consisted of one program intern, and one to two faculty veterinarians. There were generally between three and four veterinary students present on the call, however only one student was assigned as the lead on each appointment. This student would take primary responsibility during the appointment under the supervision of the program veterinarian. This included history taking, devising appropriate treatment plans, client communication, and post appointment medical record keeping. Telehealth regulations in effect during the study period allowed for establishment of a veterinary patient client relationship in order to provide a diagnosis and subsequent treatment for patients through the virtual platform. All medical records and discharges were reviewed by a program veterinarian prior to finalization. The PFL team scheduled any needed follow up appointments as directed by the Penn Vet Shelter Medicine team.

The PFL appointment interactions closely followed the service learning model within the context of higher education. Student contributions to appointments were monitored by the Penn Vet intern and faculty veterinarians in order to ensure that the experience closely matched each client's individual goals for their animals. Students were also asked to discuss their experience immediately following the end of the appointment day in order to reflect on the interaction and gain insight into its impact on the community. Penn Vet faculty instructors present during the appointment were experienced in debriefing such learning models.

Surveys were administered to clients and veterinary students through Qualtrics between January 2021 and August 2021. The survey distributed to clients was utilized to collect information on access to veterinary care, appointment outcome, and veterinary student-client communication. The survey that was distributed to fourth year veterinary students was used to comparatively evaluate client communication, appointment outcome, and the impact of these appointments on veterinary student attitudes and perspectives toward working with and providing care for the underserved. After initial review from the University of Pennsylvania's Institutional Review Board, this study was deemed minimal risk and was exempt from full review (IRB protocol number 843503).

### Client Survey

All individuals who were scheduled for a telehealth appointment with the Penn Vet Shelter Medicine Program by PFL for the first time were eligible for the study. After the conclusion of the appointment, students and faculty exited the virtual meeting space, leaving the PFL community liaison and the client on the call. The community liaison would then ask if the client would like to participate in an optional study. If the client agreed, an online survey was administered verbally. Responses to the survey were recorded anonymously by the community liaison through the Qualtrics software. The liaison was able to administer a pre-translated survey in Spanish, should the client have requested this.

The client survey included demographic data, as well as a five question survey designed to look at the effectiveness of veterinary student communication throughout the appointment from the perspective of the client. Each question was designed as a five point Likert scale from 1-5 (strongly disagree to strongly agree). There was an additional section for optional open text feedback. The client survey was administered in the same manner regardless of whether or not students were present during the appointment. Please see Table 1 for a full copy of the client survey.

**Table 1:** Client post appointment survey.

**Demographics**
*What would best describe you?*
*What is your gender?*
*What is your age?*
*What is your education level?*
*How many people live in your home including yourself (if you do not wish to answer this question, please write N/A)?*
*How many animals live in your home for the majority of the day?*
**Access to care**
*If the Pets for Life program had not provided this telehealth appointment, would you and your pet have been able to secure transportation in order to attend an appointment at an in person veterinary clinic?*
*If the Pets for Life program had not provided this telehealth appointment, would you have been able to afford an appointment at an in person veterinary clinic?*
**Comparative survey**
*Please respond to the following statements on a scale of 1–5 (strongly disagree to strongly agree):*
*My expectations for the appointment were met today*.
*I feel a strong bond with my pet*
*I understood what the vet team discussed with me today*.
*I am satisfied with the outcome of today's appointment*.
*I felt that the student involvement in the appointment was a positive experience*.
*Please provide any additional feedback regarding your experience with Pets For Life today*.

### Veterinary Student Survey

All veterinary students participating in their clinical year elective shelter medicine rotation during the study period were eligible for participation in this study. Rotations lasted 2 weeks in duration, with students participating in one PFL appointment day per week. Participation in clinical rotation activities, including telehealth appointment days, was required for a passing grade for the rotation, however completing the survey was optional and did not have any impact on grading.

The veterinary student survey portion of this study was administered to veterinary students in the form of two separate online surveys, one prior to participation in the first PFL appointments scheduled and one following participation in the final PFL scheduled appointments. At the beginning of the Shelter Medicine elective rotation, the instructors verbally described the survey, including that it would remain anonymous and their participation had no impact on their grade. Veterinary students were sent an initial pre-appointment Qualtrics survey link by the shelter medicine rotation course organizer, which included a unique ID code for each student that was used for both pre- and post-appointment surveys so that data could be paired for each individual, but anonymized. Students were able to complete this survey until their participation in the first PFL appointment during the rotation. If they did not complete the pre appointment survey prior to getting oriented to the appointments immediately preceding their participating in the first appointment, they were ineligible for the study. Approximately 48 h following the second PFL appointment day, veterinary students were sent the post appointment survey. Students were required to enter the same unique ID code that was given to them with the initial pre appointment survey. Students were sent an additional reminder email to complete the post appointment survey within the month following the conclusion of their rotation.

The pre-appointment survey included demographic information, as well as administration of the Jefferson Scale of Empathy, Medical Student Version (17). This is a 20 question validated scale that was utilized to evaluate any changes in empathy toward patients after participating in the PFL appointments. A total empathy score was calculated according to the established scoring system (17). Please see Table 2 for a full copy of the pre appointment veterinary student survey, including three items from the Jefferson Scale of Empathy for illustrative purposes.

**Table 2:** Veterinary student pre appointment survey.

**Demographics**
*What would best describe you?*
*What is your gender?*
*What is your age?*
*Have you completed your Pets for Life appointment day as part of your Shelter Medicine rotation?*
**Jefferson scale of empathy rated on a scale from 1 to 7 (strongly disagree to strongly agree).** **Three items out of 20 are listed for illustrative purposes**
*2. Patients feel better when their physicians understand their feelings*.
*7. Attention to patients' emotions is not important in history taking*.
*14. I believe that emotion has no place in the treatment of medical illness*.

The post-appointment survey included the same Likert scale questions that were administered to the client about veterinary-student client communication. In addition, the veterinary student survey included the post appointment administration of the Jefferson Scale of Empathy.

Please see Table 3 for the full veterinary student post appointment survey.

**Table 3:** Veterinary student post appointment survey.

**Demographics**
*What would best describe you?*
*What is your gender?*
*What is your age?*
*Have you completed your Pets for Life appointment day as part of your Shelter Medicine rotation?*
**Comparative survey**
*Please respond to the following statements on a scale of 1–5 (strongly disagree to strongly agree):*
*I felt my client's expectations for the appointment were met today*.
*I felt that the client had a strong bond with their animal*
*I felt that the client understood what the vet team discussed with them today*.
*I am satisfied with the outcome of today's appointment*.
*I felt that my involvement in the appointment was a positive experience for the client*.
*Please provide any additional feedback regarding your experience with Pets For Life today*.
**Jefferson scale of empathy rated on a scale from 1 to 7 (strongly disagree to strongly agree).** **Three items out of 20 are listed for illustrative purposes**.
*2. Patients feel better when their physicians understand their feelings*.
*7. Attention to patients' emotions is not important in history taking*.
*14. I believe that emotion has no place in the treatment of medical illness*.

### Statistical Analysis

Data were analyzed using SPSS Statistics (IBM SPSS Statistics for Windows, Version 27). Descriptive statistics were calculated regarding the demographic characteristics of the sample, client-student perceptions of the telehealth appointment and barriers to veterinary care. Mann Whitney U tests were used to compare client perceptions of the veterinary appointment relative to the presence of students. A Wilcoxon Signed Rank Test was used to compare pre- and post-veterinary student empathy scores. A Mann-Whitney U Test was also used to compare pre-veterinary student empathy scores between students who completed the post-survey and those who did not. Statistical significance was set at *p* < 0.05.

## Results

### Participant Demographics

A total of 31 clients and nine veterinary students were included in the final dataset. Twenty two students completed the pre appointment survey, 11 students completed the post appointment survey, and nine students had valid pre and post appointment survey responses. Of the 31 clients, 18 client responses were collected when students were present on the rotation. The remaining 13 responses were collected when only the veterinary staff was present. All 31 clients who participated in the PFL appointment days chose to participate in the study.

Clients identified as Black or African American (*n* = 9, 29%), White/Caucasian (*n* = 11, 36%), Hispanic or Latino (*n* = 9, 29%) or other (*n* = 2, 7%). Most client respondents were female (*n* = 23, 74%). The average education level possessed by clients was a high school degree or equivalent (*n* = 17, 55%). Demographic data for clients is represented in Table 4. Of the nine student participants, all of them identified as White/Caucasian female students between the ages of 25 and 34.

**Table 4:** Client demographic data.

**Characteristics**	**N**	**%**
**Race/ethnicity**		
Black or African American	9	29.0
White/Caucasian	11	35.5
Hispanic or Latino	9	29.0
Other	2	6.5
**Gender**		
Male	8	25.8
Female	23	74.2
**Age**		
18–24	3	9.7
25–34	6	19.4
35–44	6	19.4
45–54	5	16.1
Over 55	11	35.5
**Education level**		
Less than a high school diploma	2	6.5
High school degree or equivalent	17	54.8
Bachelor's degree	9	29.0
Master's degree	1	3.2
Other	2	6.5

### Client Survey Results

#### Client Perceptions

All clients agreed or strongly agreed that their expectations for the appointment were met (*n* = 5, 16% and *n* = 26, 84%, respectively). Similarly, the majority of clients agreed or strongly agreed that they understood what the veterinary team discussed with them (*n* = 1, 3% and *n* = 30, 97%, respectively). Clients also agreed or strongly agreed that they were satisfied with the outcome of their appointment (*n* = 4, 13% and *n* = 27, 87%, respectively).

Mann-Whitney U tests were utilized to compare client perception responses to each individual survey question between those who had students present and those who did not. There was no significant difference found between the responses.

#### Barriers to Veterinary Care

The majority of clients responded that they would not have been able to afford an appointment at an in person veterinary clinic had the PFL program not provided the telehealth appointment (*n* = 18, 58%). The remaining clients responded that they either would have been able to afford the appointment (16%, *n* = 5), or were not sure if they would have been able to afford the appointment (26%, *n* = 8).

The majority of clients responded that they would have been able to secure transportation to attend an in person veterinary clinic had the PFL program not provided the telehealth appointment (68%, *n* = 21). The remaining clients responded that they would not have been able to secure transportation (23%, *n* = 7) or were not sure if they would have been able to secure transportation (10%, *n* = 3). Please see Table 5 below for further representation of barriers to veterinary care data.

**Table 5:** Cross-tabulation showing overlap between transportation and cost barriers to veterinary care.

	**Transport**			
**Cost**	**Yes *n* (%)**	**No** ***n* (%)**	**Not sure *n* (%)**	**Total** ***n* (%)**
Yes *n* (%)	5 (16)	12 (39)	1 (3)	18 (58)
No *n* (%)	1 (3)	4 (13)	0 (0)	5 (16)
Not sure *n* (%)	1 (3)	5 (16)	2 (7)	8 (26)
Total *n* (%)	7 (23)	21 (68)	3 (10)	31 (100)

#### Human-Animal Bond

The majority of clients either agreed or strongly agreed that they felt a strong bond with their pet (*n* = 2, 7% and *n* = 28, 90%, respectively). The remainder of the clients were undecided (*n* = 1, 3%).

#### Student Involvement

For those appointments that included veterinary students (*n* = 18), clients agreed or strongly agreed that the student involvement in the appointment was a positive experience (*n* = 2, 11% and *n* = 16, 89%, respectively).

### Veterinary Student Survey Results

#### Veterinary Student Perceptions

Twenty-six students participated in the rotation during the study period and were eligible to complete the survey. Out of the 26 students, 22 completed the pre-rotation survey, 11 completed the post-rotation survey and nine students had both pre and post-test data. All students agreed or strongly agreed that they felt their clients expectations for the appointment were met (*n* = 7, 64% and *n* = *n* = 4, 36%, respectively). Students also agreed or strongly agreed that they felt the client understood what the veterinary team discussed with them (*n* = 7, 64% and *n* = 4, 36%, respectively). Similarly, the majority of students agreed or strongly agreed that they were satisfied with the outcome of the appointment (*n* = 7, 64% and *n* = 4, 36%, respectively).

#### Human-Animal Bond

All veterinary students agreed or strongly agreed that they felt their client had a strong bond with their animal (*n* = 2, 18% and *n* = 9, 82%, respectively).

#### Student Involvement

The majority of students agreed or strongly agreed that they felt their involvement in the appointment was a positive experience for the client (*n* = 6, 55% and *n* = 4, 36%, respectively). The remainder of the students were undecided (*n* = 1, 9%).

#### Veterinary Student Empathy

A total of nine students completed valid pre and post-test surveys. There was no significant difference in student empathy following telehealth appointments (Z = 1.55, *p* = 0.12). Veterinary students had a median total empathy score of 121 (IQR 118–133) prior to the PFL rotation and 120 (IQR 114–132) following the rotation. However, there was a significant difference in student empathy between students who completed the pre survey only and students who completed both the pre and post surveys (*U* = 93.50, *Z* = 2.34, *p* = 0.02). Students with baseline data only had a significantly lower median JSE score of 113.00 (IQR 103.50–121.50) prior to the rotation. There was no significant difference between students with post-rotation data only compared with students with valid data for both time-points (*U* = 58.00, *Z* = 0.95, *p* = 0.44).

## Discussion

There is limited research regarding the use of telehealth in veterinary medicine or veterinary student learning, and currently no research exists to evaluate its use specifically in underserved populations. This pilot study is the first of its kind to identify the role of telehealth in addressing barriers to veterinary care within an underserved population. Additionally, this is the first known study to evaluate the use of telehealth in veterinary student communication education within the context of underserved communities.

### Access to Veterinary Care

Although the majority of clients responded that they would have been able to obtain transportation to a veterinary appointment without PFL, nearly 25% of clients indicated that transportation to a veterinary appointment was a significant barrier to accessing veterinary care. This did not entirely overlap with those who could not afford care, which represents how additional modalities of care could help fill gaps in accessibility beyond just financial limitations. In a review of barriers to veterinary care among underserved populations, one of the biggest concerns for clients in terms of transportation was owners in large cities utilizing public transportation with their pets. An additional concern was veterinarians who were unwilling to open clinics in underserved areas (2). This study provides evidence in support of utilizing appropriate telehealth measures in order to identify individuals who do not have secure transportation and effectively eliminate this barrier to ultimately increase access to veterinary care.

Although this study was conducted within an urban environment, transportation barriers also exist in rural areas where access to veterinary care is geographically limited (18). Additional research looking into telehealth's ability to break down barriers such as mobility, transportation, time, and complex work schedules within different types of communities might also be effective. Although the sample size for this pilot study was limited, all of the new clients that engaged in appointments and were eligible to participate in the study opted to complete the survey. Future studies with larger sample sizes that dive deeper into the outcomes and long-term client experience could be beneficial.

The majority of clients also reported that they would not have been able to afford their appointment without the PFL program. Although this in itself does not necessarily support the use of telehealth over conventional in person veterinary appointments in increasing access to veterinary care, it seems that the initiative itself was effective at eliminating at least one barrier to care. The cost of veterinary care has historically been one of the most commonly cited reasons that owners do not seek veterinary care for their animals (2).

### Veterinary Team/Client Perceptions

Overall, survey responses from both clients and veterinary students were overwhelmingly positive. Client responses indicate that this initiative was successful in terms of client satisfaction, veterinary team communication, and student involvement. Veterinary students perceived these interactions similarly, with equally positive responses in each respective category. These responses provide support for telehealth as beneficial to both the client and the student. This also indicates that veterinary student and client perspectives were aligned on the appointment experience. Other studies evaluating veterinary student involvement in the context of service learning initiatives have found similarly positive results (13), lending similar support for such initiatives in student learning.

While the overwhelming positivity of responses provides support for such initiatives, it is also considered a limitation. Those who felt strongly about the interaction were probably more likely to provide feedback in survey form. Those who had different experiences, including more neutral or possibly even negative responses, might not have felt compelled to complete a survey regarding their experience. Therefore, it is possible that the nature of the responses could be skewed and valuable feedback from those who did not have such a positive experience was lost. However, PFL reported that all clients that were eligible to take the survey elected to and therefore negative feedback should have been captured. For the students, a much lower response rate makes this type of error more likely. Additionally, surveys were administered to clients verbally by the PFL liaison in an attempt to reduce language or technological barriers. However, the use of client interviews may have increased the risk of social desirability bias, whereby clients may have provided answers that they believed would be viewed more favorably by the liaison. Some clients may have provided more accurate information if they had the ability to self-administer the survey.

Data regarding the human-animal bond showed that both clients and veterinary students perceived a strong bond between the client and their animal. The human-animal bond has been extensively studied, with data describing a potential positive impact of pet ownership for humans on both mental (19, 20) and physical wellbeing (21), however these studies do not exclusively focus on underserved communities. Additional research suggests a complicated role of pet ownership in populations facing various forms of adversity (22). While not a major focus of this study, data from this study suggests that this bond was largely present in clients evaluated within this underserved community and was well perceived by veterinary students. Current research regarding veterinary student perception of the human-animal bond has not been extensively studied in underserved populations. One qualitative study evaluated veterinary student perception of animal welfare in the specific context of a community clinic providing care for underserved individuals. This study revealed that students had pre-existing perceptions of poor animal welfare among pets belonging to these clients, however after participating in the clinic they felt very strongly that clients did in fact share a strong bond with their pet (23). More in depth research on the perception of the human-animal bond in underserved communities by veterinary students is warranted.

The lack of significant difference in client responses between appointments with veterinary students as the primary communicator vs. appointments with program clinicians as the primary communicator suggest that appointments integrated with students in telehealth can be supervised properly to allow a positive client experience. Although having multiple individuals on a telehealth call had the potential to be confusing or distressing to clients, this did not seem to change their perceptions of the appointment experience. More exploration on how to create properly supervised experiences that protect the target community's experience and quality of care and that can assist with student learning is critical as these new modalities and community interventions are developed. In the clinical setting, whether in telehealth or more traditional in-person clinical settings, properly developing programming with students including careful assessment is critical to ethical community engagement. Training future practitioners in how to perform telemedicine and telehealth appointments will also likely be important skills to prepare them for the future of the veterinary field.

### Veterinary Student Empathy

The Jefferson Scale of Empathy utilized in this study did not show any significant difference in empathy after students completed their PFL appointment days, which could be due to several reasons. To the author's knowledge, there has only been one other study that utilizes the Jefferson Scale of Empathy within the context of veterinary medicine (24). As this scale was originally developed to evaluate medical students specifically within the context of human medicine, it is possible that some of the questions did not adequately translate well into the veterinary setting. The largest limitation, both in regards to the scale and throughout the study, was sample size. The small number of responses represented only one demographic (White females between the ages of 25 and 34) and might not accurately reflect the views of the majority of veterinary students. However, it is interesting to note that White females currently comprise the majority of the present day veterinary student population, so this data might actually be representative of the field to some degree (25). Out of 26 students who participated in the rotation during that time period, 22 elected to fill out the pre-test, however, only 11 completed the post-test survey. Students who completed the pre-test only had significantly lower empathy scores prior to the rotation than students who had valid data at both time points, which may have contributed to the null findings pertaining to student empathy relative to the rotation. For example, it is possible that a ceiling effect occurred, whereby students with both pre and post data already had high levels of empathy and thus, did not report a measurable increase in empathy following the rotation. Future research with more veterinary students, including students who do not report high levels of empathy initially, could help elucidate more nuanced changes in student learning.

Overall, median scores on the Jefferson Scale of Empathy for veterinary students were similar to previously published estimates in females (26), with no significant difference in pre and post appointment scores. It is possible that students had previous opportunities to engage with underserved communities either within or outside of the context of veterinary medicine. The rotation itself is elective, which might self-select for those students who are more informed about community work. Due to its strong emphasis on the community and other curricular electives offered by the shelter medicine program integrating these concepts, students might already have been exposed.

Lastly, the PFL appointments comprised a total of only several hours over 2 weeks. The limited interaction time paired with a short period between pre and post scale administration could be contributing to the lack of significant difference. Students who participate in longer term initiatives with more client interaction might be more likely to be impacted by their experiences and reveal a significant increase in empathy toward clients. However, a previously unpublished study on journaling on this rotation found that these PFL appointments were highly valued and represented strong development of understanding of the community (Jafarian et al., manuscript in preparation). It is possible that the Jefferson Scale of Empathy was unable to capture the nuances more evident in qualitative analysis, such as in the previously described study. It is also possible that this scale might not have been entirely applicable to veterinary medical students, as it was originally designed for use in human medicine. Further investigation into experiences that shape veterinary student empathy including mapping student experiences and perceptions before and after interventions is warranted.

## Conclusions

This study provides a preliminary report on the impact of targeted initiatives for underserved communities involving veterinary students within the context of a telehealth appointment. The feedback from both clients and veterinary students implies that these interactions were overall positive. Veterinary students were able to integrate into appointments while maintaining clear communication and accomplishing client goals. Further investigation is needed to fully evaluate the scope of technical and interpersonal skills gained by veterinary students within this context. Barriers to veterinary care were identified and addressed through this study and provide support for identification of additional initiatives that can continue to increase access to care. The use of telehealth specifically proves to be an emerging but promising means of providing basic care to underserved communities. Additional research is needed to assess its role within a larger population across a wider variety of applications.

## Data Availability Statement

The datasets presented in this article are not readily available because data governance arrangements limit sharing the full dataset. Requests to access the datasets should be directed to brittawa@vet.upenn.edu.

## Ethics Statement

This study went through an initial review from the University of Pennsylvania Institutional Review Board and was deemed a minimal risk study. It was therefore exempt from full review. Veterinary student participants provided informed consent electronically. Clients were verbally asked by the PFL community liaison if they consented to participation in an optional survey. The patients/participants provided their written informed consent to participate in this study.

## Author Contributions

LL, BW, and CR contributed to conception and design of the study. EH oversaw data collection. LP performed the statistical analysis and data visualization. LL wrote the first draft of the manuscript. BW, CR, and LP provided feedback and contributed to editing the manuscript. All authors contributed to manuscript revision, read, and approved the submitted version.

## Funding

This project was funded by the Penn Vet Rosenthal Shelter Medicine Fellowship. Salary for CR was provided by grant funding from the Bernice Barbour Foundation, and salary for EH and LP was provided by grant funding from the Arnall Family Foundation. The telehealth service of the Penn Vet Shelter Medicine Program was developed as a project for the Penn Vet WaterShed Community Medicine Internship, funded by the Arnall Family Foundation.

## Conflict of Interest

EH was employed by Philadelphia Animal Welfare Society. The remaining authors declare that the research was conducted in the absence of any commercial or financial relationships that could be construed as a potential conflict of interest.

## Publisher's Note

All claims expressed in this article are solely those of the authors and do not necessarily represent those of their affiliated organizations, or those of the publisher, the editors and the reviewers. Any product that may be evaluated in this article, or claim that may be made by its manufacturer, is not guaranteed or endorsed by the publisher.
